# Physicochemical and Biological Performance of Aloe Vera-Incorporated Native Collagen Films

**DOI:** 10.3390/pharmaceutics12121173

**Published:** 2020-12-02

**Authors:** Mireia Andonegi, Ainhoa Irastorza, Ander Izeta, Koro de la Caba, Pedro Guerrero

**Affiliations:** 1 BIOMAT Research Group, University of the Basque Country (UPV/EHU), Escuela de Ingeniería de Gipuzkoa, Plaza de Europa 1, 20018 Donostia-San Sebastián, Spain; mireia.andonegui@ehu.eus; 2 Tissue Engineering Group, Biodonostia Health Research Institute, Paseo Doctor Begiristain s/n, 20014 Donostia-San Sebastián, Spain; ainhoa.irastorza@biodonostia.org (A.I.); ander.izeta@biodonostia.org (A.I.)

**Keywords:** native collagen, aloe vera, films, biomaterials

## Abstract

Collagen was obtained from porcine skin by mechanical pretreatments with the aim of preserving the triple helix structure of native collagen, which was indirectly corroborated by differential scanning calorimetry (DSC) and Fourier transform infrared spectroscopy (FTIR) results. Moreover, aloe vera (AV), with inherent biological properties, was incorporated into collagen film formulations, and films were prepared by compression and characterized to assess their suitability for biomedical applications. SEM images showed that the fibrillar structure of collagen changed to a rougher structure with the addition of AV, in accordance with the decrease in the lateral packaging of collagen chains observed by XRD analysis. These results suggested interactions between collagen and AV, as observed by FTIR. Considering that AV content higher than 20 wt % did not promote further interactions, this formulation was employed for biological assays and the suitability of AV/collagen films developed for biomedical applications was confirmed.

## 1. Introduction

Many polymers derived from biomass have been widely evaluated for biomedical applications, such as tissue engineering, drug delivery and wound healing, among others, in order to provide a green alternative to avoid the employment of toxic solvents and non-biodegradable polymers [1]. Among natural polymers, proteins such as collagen [2] and polysaccharides such as chitosan [3] may be very valuable due to their intrinsic bioactivity, biocompatibility and biodegradability [4]. When these materials are located in a defective area, they may promote the migration of cells and their integration to the surrounding environment, thus forming an extracellular matrix (ECM) and improving the structure of the repaired tissue [5].

In this context, collagen is one of the most important ECM proteins and it is considered as a potential biomaterial for tissue engineering [6]. Collagen consists of three polypeptide chains organized in a triple helical conformation. This structure is shaped into more complex ones, leading to at least 29 collagen types in which type I, II and III collagens are the most abundant types [7]. Among them, collagen I shows a more ordered structure and, thus, provides higher tensile strength, being used for tissue regeneration [8,9]. In fact, collagen is the most widespread protein in connective tissue, and it plays an important role as a natural binding segment in tissue healing, providing the biological microenvironment needed for cell adhesion, proliferation and migration during the tissue construction or repair process [10]. Besides its biological properties, collagen also contains abundant intra- and intermolecular hydrogen and disulfide bonds and, therefore, inherent water stability. Collagen can be extracted from almost every living animal; nonetheless, common sources of collagen include bovine skin and tendons, porcine skin, and rat-tail, although it is noteworthy that porcine skin is the most similar to human skin [11]. Due to collagen properties, abundance and ease of extraction, the research on collagen-based materials is growing intensively [12]. Of note, collagen can be manufactured into films [13], hydrogels [14], micro/nanoparticles [15] and porous structures [16] for biomedical applications [17].

Likewise, the mucilaginous gel of aloe vera (*Aloe barbadensis*) plant has been used as a natural therapeutic agent for medical purposes in several cultures for millennia due to its physiologically active components with curative or healing qualities [18]. In traditional medicine, for example, aloe vera has been used to cure various types of soft tissue damages, such as skin diseases, injuries caused by irradiation, eye diseases, intestinal disorders and viral diseases [19]. Aloe vera gel includes more than 98–99% water and 55% of its dry matter is a glucomannan polysaccharide known as acemannan, which stimulates cell proliferation and has antiviral, anticancer and immune stimulation effects [5,20]. Additionally, aloe vera contains sugars, essential amino acids, lipids, sterols and a variety of vitamins [21] and it has been reported to enhance cell proliferation and wound care [22], providing antioxidant, anti-inflammatory, antibacterial, antidiabetic, sunburn relief, immune boost, anti-ageing, and anticancer properties [23,24]. For these reasons, aloe vera is applied to a variety of products in the form of juice, concentrate and powder for several pharmaceutical, food, cosmetic and biomedical applications. Pharmaceutical use of aloe vera includes tablets, capsules and ointment due to its effectivity for wound healing [25], genital herpes [26], and HIV infection [27], and also owing to anti-ulcer effects [28], anti-inflammatory properties [29], and antidiabetic effects [30]. In particular, the latest research studies on aloe vera are focused on wound healing, especially related to burns. To this end, biomaterials combining aloe vera with various biopolymers, such as cellulose, alginate, chitosan, collagen or gelatin, and functional films, hydrogels and sponges, have been developed [19,31,32,33]. These biomaterials, in comparison with conventional products, offer several properties providing mechanical support, prolonged drug release, absorption of skin secretions and preservation of body fluids [34].

Regarding the film-forming process, solution casting is the most used method to obtain collagen films. This process is based on the solubilization of collagen in an acidic and/or enzymatic aqueous media and the subsequent evaporation of the solvent [35]. In contrast, compression molding is a faster and more efficient method; thus, the processing technique used at industrial scale production [6]. Therefore, in this study, thermo-compression was used to revalorize porcine skin, transforming it on collagen-based films, and the effect of the incorporation of aloe vera on physicochemical, thermal, barrier, mechanical and biological properties was analyzed.

## 2. Materials and Methods

### 2.1. Materials

Porcine collagen was supplied by Tenerias Omega (Villatuerta, Spain) and aloe vera (AV) powder by Agora Valencia (Valencia, Spain). Glycerol, pharma grade with a purity of 99.01%, and acetic acid were supplied by Panreac Quimica S.L.U (Barcelona, Spain).

### 2.2. Mixture Preparation

Native collagen was obtained from porcine skin through the method described in a previous work [36]. Briefly, porcine skins were immersed into 1 M NaOH solution at room temperature for 12 h in order to be defatted. Then, these samples were neutralized by immersion into phosphate buffer saline (PBS) at pH 7.4 for 3 h. Finally, samples were grinded and lyophilized. After these treatments, 5 g of collagen, aloe vera (0, 10, 20 and 30 wt % based on dry collagen), glycerol (20 wt %, based on dry collagen) and 0.05 M acetic acid (1:1 collagen/acetic acid ratio) were manually mixed and pastes were stored in a plastic bag for 24 h at room temperature for dough hydration. In total, 10, 20 and 30 wt % aloe vera contents were selected considering that AV acts as a plasticizer and, thus, facilitates the flow of mixtures when processed by compression molding. Four film systems were produced and designated as control, AV10, AV20 and AV30, as a function of aloe vera content.

### 2.3. Film Processing

The hydrated dough was molded in a hot press. The dough was placed between two aluminum plates, put into a Specac press, previously heated up to 90 °C for 30 s, and then pressed at 0.5 MPa for 1 min to obtain the films. It is worth noting that those temperature and pressure conditions were selected since no film could be obtained at lower temperatures and pressures. All samples were conditioned in an ACS Sunrise 700 V bio-chamber (Alava Ingenieros, Madrid, Spain) at 25 °C and 50% relative humidity for 48 h before testing. 

### 2.4. Fourier Transform Infrared (FTIR) Spectroscopy

Fourier transform infrared (FTIR) spectroscopy was performed by using a Nicolet 380 FTIR spectrometer (Nicolet Instrument, Barcelona, Spain) employing an attenuated total reflectance (ATR) crystal (ZnSe). In total, 32 scans were carried out and the resolution used was of 4 cm^−1^.

### 2.5. Moisture Content (MC) and Mass Loss (ML)

Films were weighed (w_0_), lyophilized and reweighed (w_1_) to calculate MC values as:

(1) $$\mathrm{MC}\;\left(\%\right)=\frac{w_{0}-w_{1}}{w_{0}}\times \;100$$

Dried films (w_1_) were immersed into PBS (pH 7.4) for 5 days, and then reweighed (w_2_) to calculate ML values as:

(2) $$\mathrm{ML}\;\left(\%\right)=\frac{w_{1}-w_{2}}{w_{1}}\times \;100$$

### 2.6. Water Uptake (WU)

Films were cut into rectangular pieces of 1 cm × 2 cm, weighed (w_i_), submerged into PBS (pH 7.4), and reweighed at specific times (w_f_) until constant values. WU was determined by the following equation:

(3) $$\mathrm{WU}\;\left(\%\right)=\frac{w_{f}-w_{i}}{w_{f}}\times \;100$$

### 2.7. Thermo-Gravimetric Analysis (TGA)

A Mettler Toledo TGA/SDTA 851 thermo-balance (Mettler Toledo, Barcelona, Spain) was used to determine the film thermal stability. Measurements were performed from −25 to 750 °C at 10 °C/min and nitrogen atmosphere (10 mL N_2_/min) was used to avoid thermo-oxidative reactions.

### 2.8. Differential Scanning Calorimetry (DSC)

A Mettler Toledo DSC 822 (Mettler Toledo, Barcelona, Spain) was employed to carry out DSC measurements. Samples (3 mg) were heated from −50 to 250 °C at 10 °C/min using nitrogen atmosphere to avoid oxidation and sealed aluminum pans to prevent mass loss. Additionally, control and AV20 samples were subjected to a two-heating ramp at the same conditions, firstly from 25 to 125 °C and then from 25 to 250 °C.

### 2.9. Ultraviolet–Visible (UV–Vis) Spectroscopy

A UV-Jasco V-630 spectrophotometer (Jasco, Barcelona, Spain) was used to measure the light absorption from 200 to 800 nm.

### 2.10. Water Contact Angle (WCA)

WCA values were measured using a DataPhysics OCA 20 (Neurtek, Eibar, Spain) system. The water droplet (3 μL) was placed on the film surface, and the WCA value was determined using SCA20 software to capture the drop image.

### 2.11. Mechanical Properties

Bone-shaped samples (4.75 mm × 22.25 mm) were cut and an Instron 5967 electromechanical testing system (Instron, Barcelona, Spain) was used to carry out tensile tests at 1 mm/min, according to ASTM D 638-03 standard. 

### 2.12. X-ray Diffraction (XRD)

XRD measurements were performed at 40 kV and 40 mA with a diffraction unit (PANalytical Xpert PRO, Madrid, Spain), generating the radiation from a Cu-Kα (λ = 1.5418 Å) source. Data were recorded from 2° to 50°.

### 2.13. Scanning Electron Microscopy (SEM)

Films were placed on a metal stub and coated with gold using a JEOL fine-coat ion sputter JFC-1100 and argon atmosphere. Samples were observed using a Hitachi S-4800 scanning electron microscope (Hitachi, Madrid, Spain) at 15 kV accelerating voltage.

### 2.14. Degradation Studies

Degradation studies were performed in order to determine the weight loss due to the hydrolytic, cell-mediated and “simulated body fluid” degradation. For this purpose, the material was cut into 8 mm diameter discs and weighed (*W*_0_). Following this, samples were exposed to the three different degradation agents (phosphate buffer saline (PBS, pH 7.4), cells in complete medium, and fetal bovine serum (FBS)), and incubated at 37 °C. At different time-points, the samples were removed, freeze-dried and weighed (*W*_t_). The degradation degree (*DD*) was calculated with the following equation:

(4) $$DD\;\left(\%\right)=\frac{W_{0}-W_{t}}{W_{0}}\;\times \;100$$

Every test was performed in five-fold, with average values obtained for each time point (day 1, 4, 7, 14, 21 and 28) and charted versus time. The hydrolytic degradation (HDD) was performed submerging the scaffolds into 500 µL of PBS. For cell-mediated degradation (CDD) 30,000 cells of HS27 fibroblasts scaffold were seeded per scaffold. To assess the “simulated body fluid” degradation (BFDD) of the biomaterials, 500 µL of FBS was added into the well.

### 2.15. Cell Culture

Following the recommendations of the ISO 10993-5:2009 guidelines for biological evaluation of medical devices, HS27 (ECACC) cells were cultured on complete medium that was composed of Dulbecco’s modified Eagle’s medium (Sigma) supplemented with 10% (*v*/*v*) inactive FBS (Sigma), 1% (*v*/*v*) penicillin-streptomycin (Lonza) and 1% (*v*/*v*) L-glutamine (Gibco) at 37 °C in a humidified incubator with a 5% CO_2_ atmosphere. All the biological assays were carried out in aseptic conditions and the cell passages were performed weekly depending on the cell confluence. At the fifth passage HS27 cells were collected by 0.05% trypsin (Sigma, Darmstadt, Germany) and centrifuged at 1500 rpm for 5 min at room temperature. The obtained pellet was resuspended in the previously described medium to obtain a homogeneous cell suspension, after which the experimental plates of the degradation and biocompatibility studies were carried out.

### 2.16. Biocompatibility Assay

For the biocompatibility assessment, 10,000 cells/cm^2^ were seeded into a 24 well plate in 200 µL of complete medium. After 24 h of culture at 37 °C and 5% CO_2_ atmosphere, samples were placed in contact with the cells. In accordance with the ISO 10993-5:2009 standard, a distinction was made between indirect and direct exposure to the material, by placing the samples on transwells or immediately on top of the cells, respectively. The scaffolds were previously sterilized by submerging in 70% ethanol for 10 min, UV for 30 min, and washed by dialysis in PBS (pH 7.4) for 72 h. Additionally, some wells were left without biomaterial to be included subsequently as positive and negative controls. All wells were supplemented with 300 µL of culture medium. The following assays were performed in order to evaluate the biocompatibility of HS27 cells under the exposure of the film based on porcine collagen and 20% aloe vera.

On the one hand, cell mortality was assessed according to plasma membrane integrity. The Cell-Tox Green Cytotoxicity Assay (Promega #G8742) was used for this purpose. To this end, the protocol recommended by the manufacturer was followed. Briefly, the dye was added to the different wells by diluting it with the medium in a ratio of 1:1000 and incubated for 15 min protected from light, after which the fluorescence was measured.

On the other hand, metabolic activity was measured based on the reductive capacity of the cells. In this case, the Cell Counting Kit-8 colorimetric assay (Merck #96992) was used. Following the manufacturer’s recommendations, and after several washes with PBS 1× (pH 7.4) to remove the products from the previous assay, the compound was added and after 2 h of incubation the absorbance was measured.

In addition to the study conditions, positive and negative controls were included. For the first ones (CTR+), cells seeded in the same conditions but without biomaterial were used. For the negative controls (CTR-), cells treated with the lysis buffer provided by Promega’s kit were used to cause cell death. Assays were conducted at exposure days 1, 2, 4, and 7, with each condition being analyzed in triplicate. Once the results were obtained, they were relativized to the controls. In the case of mortality, the CTR- was considered as 100% of mortality, and 0% the CTR+; while in the assay of metabolic activity they were considered inversely.

### 2.17. Statistical Analysis

Results were expressed as mean ± standard deviation. Tukey’s test with a statistical significance at the *p* < 0.05 level was considered for multiple comparisons among different systems. All the statistical computations were assessed using SPSS 25.0 (IBM, Madrid, Spain, 2017).

## 3. Results and Discussion

### 3.1. Physicochemical Properties

**Figure 1:**
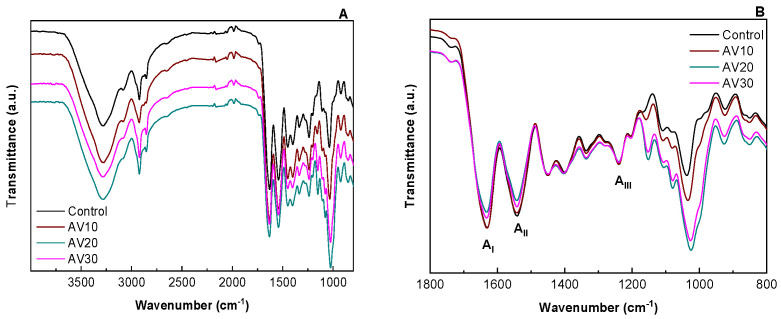
FTIR spectra of aloe vera (AV)/collagen films: (**A**) from 4000 to 800 cm^−1^ and (**B**) from 1800 to 800 cm^−1^.

In order to assess the interactions among the components of the film forming formulation, FTIR spectra of collagen films are shown in Figure 1. The broad band at around 3000–3600 cm^−1^ (Figure 1A) can be attributed to the stretching of hydroxyl groups of uronic acid, mannose, and galacturonic acid, and to phenolic groups in antraquinones present in AV, as well as to amide A (N-H stretching) in collagen [37,38]. Additionally, all the spectra showed the characteristic absorption bands assigned to the peptide bonds in collagen (Figure 1B): 1630 cm^−1^ for amide I (C=O stretching), 1542 cm^−1^ for amide II (N-H bending), and 1240 cm^−1^ for amide III (C-N stretching). The band around 1630 cm^−1^ can also be assigned to the stretching vibrations of carbonyl groups in aloe vera [37] and the shoulder at 1245 cm^−1^ could be due to the C-O-C stretches of acetyl groups, which may indicate the presence of storage bioactive polysaccharides, such as acemannan and glucamannans [39]. The spectral region between 1200 cm^−1^ and 900 cm^−1^ is attributed to the stretching vibrations of C-O bonds in collagen, those related to hydroxyl groups in glycerol, as well as those associated to polysaccharides and sugars in aloe vera [36]. The changes in the relative intensity between bands in this area suggest that the interactions among the components of the film forming formulation are physical bonds, mainly among carboxyl, amino, and hydroxyl groups present in collagen, glycerol, and aloe vera. In particular, the band at 992 cm^−1^ corresponds to the stretching of C-O bonds of hemicellulose, pectin, and lignin present in AV, and the band at 1037 cm^−1^ is associated to C-O vibrations in collagen. With the addition of AV, the band corresponding to C-O bonds shifted to 1034 cm^−1^ for 10 AV, to 1026 cm^−1^ for 20 AV, and to 1025 cm^−1^ for 30AV. Therefore, it can be said that AV contents higher than 20 wt % did not cause further shifting and did not promote further interactions with collagen and glycerol.

The moisture content (MC) and the mass loss (ML) of the films were measured and values are shown in Table 1. As can be seen, MC values decreased from 12.2% in control films to 5.8% in AV30 due to the interactions among polar groups, reducing the affinity of the films for moisture. In contrast, ML values increased for AV-incorporated films. In particular, the ML value around 20% in control films is related to glycerol and the increase in this value for the films with AV can be related to the dissolution of AV [40].

**Figure 2:**
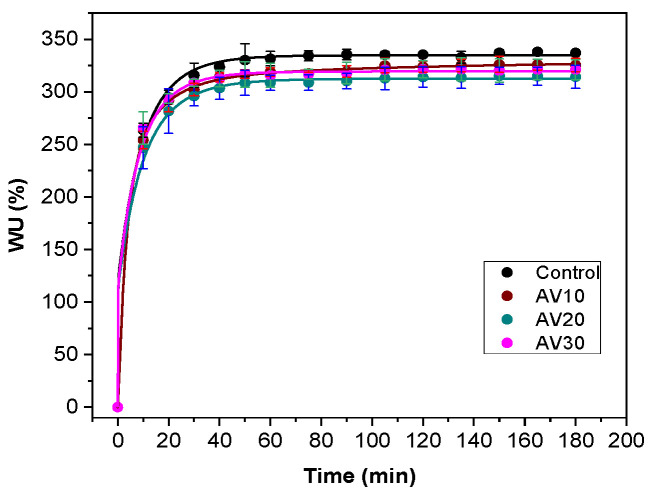
Water uptake (WU) capacity of aloe vera (AV)/collagen films.

Considering the potential application of these composite films as wound dressings, swelling tests were carried out to determine the capacity of the films to absorb exudates from wounds. As can be seen in Figure 2, all films present a rapid absorption of water along the first 20 min of immersion in PBS (pH 7.4), reaching the equilibrium in approximately 40 min with WU values around 300%, regardless of AV content. These results indicated that the addition of AV did not affect the water uptake capacity of collagen films, a required property for wound dressing purposes.

### 3.2. Thermal Properties

**Figure 3:**
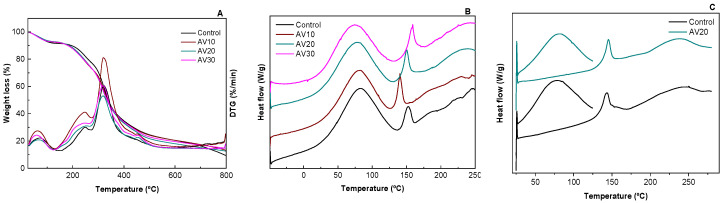
Thermal properties of aloe vera (AV)/collagen films: (**A**) thermo-gravimetric analysis (TGA) and DTG curves and differential scanning calorimetry (DSC) curves (**B**) for a single scan and (**C**) for two scans.

The thermal behavior of the films was tested by TGA and DSC analyses. TGA and DTG curves of collagen films are displayed in Figure 3A. All films showed similar behavior with three main stages. For temperatures below 150 °C, the weight loss was attributed to the loss of adsorbed and bound water, which was around 10%. The second stage, at 240–250 °C, was ascribed to the glycerol evaporation which is higher than the boiling temperature of glycerol (182 °C), indicating the interactions of the hydroxyl groups of glycerol with the polar groups of collagen and AV, in accordance with FTIR results. When AV content increased, the DTG curve became broader and this second stage can also be related to the degradation of the hemicellulose and sugars present in AV [22,41]. The third stage, for temperatures above 270 °C, was referred to the thermal degradation of collagen [42] and aloe vera [41]. Finally, the slight weight loss at 453 °C in the films with AV is attributed to the thermal degradation of cellulose and lignin present in AV [43].

The effect of compression temperature and AV addition on the denaturation of collagen was analyzed by DSC and the results are shown in Figure 3B. All samples exhibited two endothermic peaks: the first peak, around 80 °C, was related to free water release, and the second peak at around 150 °C was associated to denaturation of the collagen triple helix [44]. These values are in accordance with those obtained for bovine skin collagen [45]. It is worth noting that the denaturation peak also appeared when AV was added, indicating maintenance of the triple helix structure after AV addition and compression molding. Additionally, for a better analysis of the collagen denaturation peak, the possible interferences caused by the evaporation of water were avoided subjecting selected samples to a heating ramp to eliminate moisture. As can be seen in Figure 3C, no difference was observed in the denaturation peak with the addition of AV, confirming the prevalence of the triple helix structure when AV was added.

### 3.3. Barrier and Mechanical Properties

**Figure 4:**
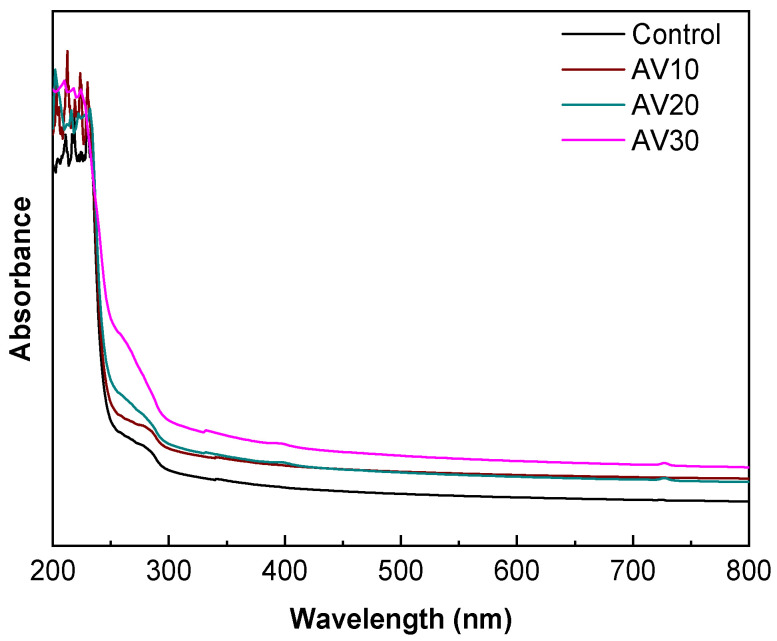
UV–vis spectra of aloe vera (AV)/collagen films.

Light barrier properties of the films were analyzed, and UV–vis spectra are shown in Figure 4. Films exhibited a small absorbance peak between 250 and 280 nm associated to aromatic amino acids residues on collagen, such as phenylalanine and tyrosine, and a maximum absorbance peak from 200 to 240 nm related to carbonyl groups present in collagen [12,46]. Similar values, around 230 nm, were found for type I fish collagen [47]. The addition of aloe vera increased the absorbance in the 250–290 nm range, attributed to the flavonoids present in AV [48]. Therefore, considering that one potential application of collagen films could be as wound dressing, the addition of AV may provide higher UV-light resistance during wound care.

Furthermore, the film hydrophilic character was analyzed by the measurement of water contact angles. As shown in Table 2, WCA values significantly (*p* < 0.05) decreased from 107° to 86° by the incorporation of aloe vera, leading to hydrophilic surfaces. These values are in agreement with WCA values observed for collagen films for regenerative applications [49]. This increase in hydrophilic character of the films can allow better draining of excess secretions in wounds. As also displayed in Table 2, mechanical properties were also influenced by the addition of aloe vera. It was found that tensile strength slightly decreased when aloe vera was added, regardless of AV concentration. In the same manner, Young modulus was not significantly (*p* > 0.05) affected by the concentration of AV. Regarding elongation at break, EAB values decreased, especially for the films with the highest content of AV, probably due to the rigid structure of AV, which limits the chain mobility.

### 3.4. Morphological Properties

**Figure 5:**
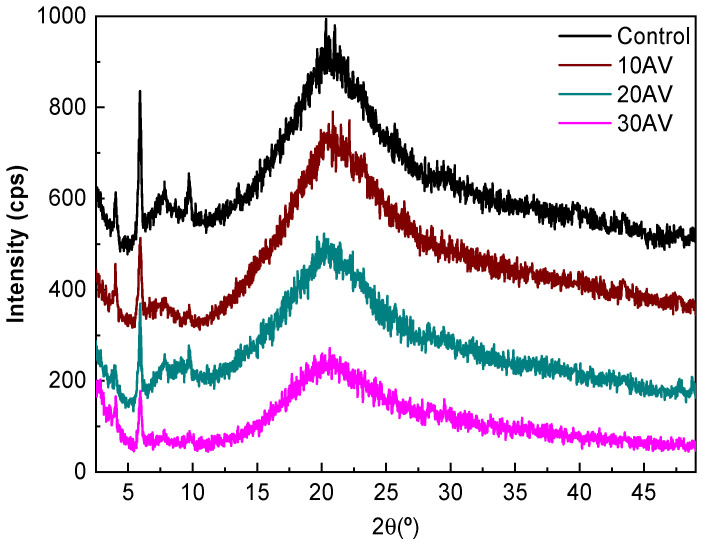
XRD patterns of aloe vera (AV)/collagen films.

In order to investigate the film structure and relate it to the properties measured, XRD and SEM analyses were carried out. As can be seen in Figure 5, films exhibited an XRD pattern characteristic of partially crystalline materials, with a small peak around 2θ = 7°, indicating an intermolecular lateral packing distance between the molecular collagen chains, and a broad diffuse peak at about 2θ = 20° due to the diffuse scattering of collagen fibers, representing the amorphous structure of the films [50]. These peaks were also found for fish skin collagen [51]. The intensity of the peaks at 7° and 20° slightly decreased with the addition of aloe vera, suggesting the decrease in the structural order.

**Figure 6:**
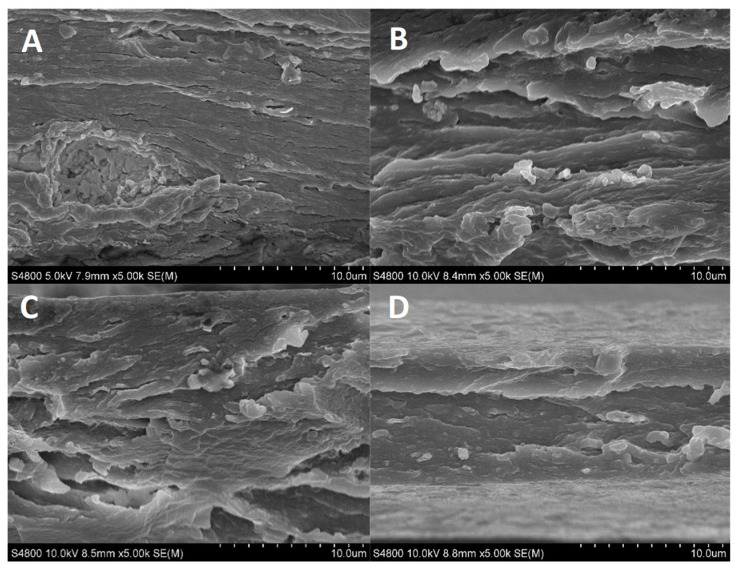
SEM images for the cross-sections of (**A**) control, (**B**) AV10, (**C**) AV20 and (**D**) AV30 films.

To further assess the effect of aloe vera on collagen structure, SEM analysis was carried out and the images of cross-sections are shown in Figure 6. All films showed a compact and homogeneous structure. Control films exhibited a dense fibrillar structure and this structure changed to a rougher structure with the addition of aloe vera, in accordance with the decrease in the lateral packaging of collagen chains observed by XRD analysis.

### 3.5. Degradation and Biocompatibility Studies

**Figure 7:**
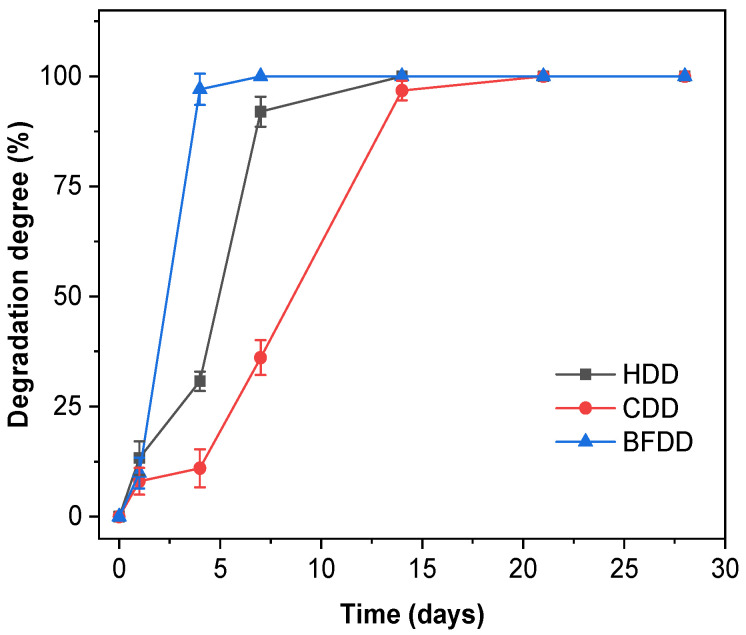
Hydrolytic (HDD), cell-mediated (CDD) and “simulated body fluid” (BFDD) degradation degrees for AV20 films.

Degradation studies were conducted to evaluate the behavior of biomaterials exposed to PBS (pH 7.4), “simulated body fluid” and cell suspension (Figure 7). With respect to hydrolytic degradation degree (HDD), films suffered a degradation of 13.29% ± 0.86 on the first day, rising until day 7 when complete degradation was achieved. Similarly, cell-related degradation (CDD) began with a DD of 8.05% ± 1.05 by day 1, increased to 36.10% ± 1.96 by day 7, and peaked at day 14. Finally, the films under the exposure of FBS as “simulated body fluid” (BFDD) underwent a slight degradation (9.90% ± 1.30) after 24 h, and then suffered a drastic degradation at day 4. These results are consistent with the literature as collagen biomaterials are known for their high biodegradation ability [52].

**Figure 8:**
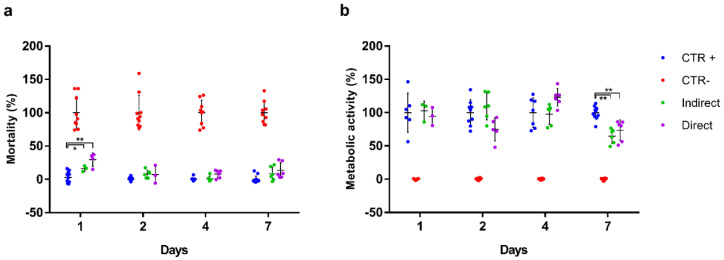
Biocompatibility assessment of the collagen and aloe vera scaffold on HS27 cells at days 1, 2, 4 and 7 of culture. Results are relativized to the controls. (**a**) Cell mortality based on the CellTox Green Cytotoxicity assay. (**b**) Metabolic activity based on the CCK-8 test. Asterisks indicate significant differences between the samples and the positive control (* *p* < 0.05, ** *p* < 0.01).

Concerning the biocompatibility assessment (Figure 8), after 24 h from the placement of the material, the cells showed a behavior related to the exposure to the sample and the release of possible excipients [53]. A statistically significant, transient mortality was observed with respect to the positive control in both the indirect and direct assays (16.1 ± 4.4% and 29.5 ± 10.0%, respectively). After 48 h, cell mortality decreased considerably, reaching acceptable values for a cell culture to the last day of study. With regard to metabolic activity, cells showed optimal values from the first day (102.6 ± 5.8% and 94.6 ± 13.6%, respectively), reaching the maximum activity at 2 days in case of the indirect exposure and 4 days of culture in case of the direct one. By day 7, a decrease in cellular metabolic activity (64.7 ± 10.9% and 73.7 ± 15.6%, respectively) was observed and attributed to the confluence of the culture and the shortage of nutrients (media were not changed through the duration of the experiments). 

In summary, the biomaterial composed of porcine collagen and aloe vera could be considered biocompatible for the cells studied. According to ISO 10993-5: 2009, the biomaterial reaches 70% viability in both indirect and direct tests, at all study times, (except for indirect exposure on day 7) with no remarkable mortality data once the culture is established. These results were expected since collagen biocompatibility is well established [52].

## 4. Conclusions

Collagen films were prepared with aloe vera by compression molding with the aim of enhancing functional properties for biomedical applications. The addition of AV increased the hydrophilic character of the surface, which allows a better fibroblast adhesion during wound healing, thus promoting tissue regeneration. Furthermore, the water uptake behavior of collagen films was not modified, and the film integrity was preserved after immersion. This performance was explained by XRD and SEM results, which showed the prevalence of the triple helix structure of native collagen with a slight decrease in the structural order with the addition of AV. Additionally, collagen films were easy to handle and showed good mechanical properties, with a slight decrease in tensile strength when AV content increased, probably due to the decrease in the structural order observed by XRD analysis. Furthermore, FTIR analysis indicated that AV contents higher than 20 wt % did not promote further physical interactions between collagen and aloe vera and, thus, this was the composition selected to confirm the suitability of aloe vera-incorporated collagen films for biomedical applications.

## Figures and Tables

**Table 1:** Moisture content (MC) and mass loss (ML) of aloe vera (AV)/collagen films.

Film	MC (%)	ML (%)
Control	12.2 ± 0.7 ^a^	21.9 ± 0.7 ^a^
AV10	9.8 ± 0.4 ^b^	31.1 ± 0.8 ^b^
AV20	7.3 ± 0.4 ^c^	31.7 ± 0.5 ^b^
AV30	5.8 ± 0.6 ^c^	35.6 ± 0.8 ^c^

^a–c^ Two means followed by the same letter in the same column are not significantly (*p* > 0.05) different through the Tukey’s multiple range test.

**Table 2:** Water contact angle (WCA), Young modulus (YM), tensile strength (TS), and elongation at break (EB) of aloe vera (AV)/collagen films.

Film	WCA (°)	YM (MPa)	TS (MPa)	EAB (%)
Control	107 ± 5 ^a^	805 ± 25 ^a^	13.8 ± 1.1 ^a^	13.5 ± 1.0 ^a^
AV10	106 ± 3 ^a^	840 ± 11 ^a,b^	11.5 ± 0.8 ^b^	11.5 ± 1.2 ^b^
AV20	97 ± 3 ^b^	867 ± 11 ^a,b^	11.3 ± 1.3 ^b^	11.2 ± 0.8 ^b^
AV30	86 ± 5 ^c^	879 ± 52 ^a,b^	10.2 ± 0.7 ^b^	8.6 ± 0.8 ^c^

^a–c^ Two means followed by the same letter in the same column are not significantly (*p* > 0.05) different through the Tukey’s multiple range test.
